# Low-dose colchicine for recurrent pericardial effusion in elderly patients: Case reports and literature review

**DOI:** 10.1097/MD.0000000000044470

**Published:** 2025-09-19

**Authors:** Yang-Jian Zheng, Wen-Na Wang, Hai-Ying Lu, Peng-Ju Yan, Shan-Kuan Liu

**Affiliations:** a Department of Cardiology, Putuo Hospital, Zhoushan, Zhejiang Province, China; b Department of Gynecological Endocrinology, Putuo Hospital, Zhoushan, Zhejiang Province, China.

**Keywords:** cardiac tamponade, case reports, elderly patients, literature review, low-dose colchicine, recurrent pericardial effusion

## Abstract

**Rationale:**

Recurrent pericardial effusion increases patient discomfort and the frequency of hospital readmissions. As effusion progresses, patients may experience cardiac tamponade, a condition where fluid builds up around the heart, making it difficult for the heart to pump blood, which can be life-threatening. In elderly patients, managing recurrent pericardial effusion can be challenging owing to multiple comorbidities. Reports on the efficacy of low-dose colchicine in elderly patients with recurrent pericardial effusion are limited. This paper presents 3 cases of low-dose colchicine treatment for recurrent pericardial effusion in elderly patients (≥80 years) and reviews the literature.

**Patient concerns:**

The first patient (Case 1) was hospitalized several times due to recurrent shortness of breath and edema. The second patient (Case 2) presented with complete atrioventricular block as a complication of acute ST-segment elevation myocardial infarction (inferior wall) and was experiencing recurrent pericardial effusion related to perforation of the electrode after removal of the temporary pacemaker. The third patient (Case 3) presented with a rare case of pericardial effusion characterized mainly by dizziness and syncope.

**Diagnoses:**

These 3 cases of pericardial effusion were seen at Putuo Hospital within the past year. Moderate to severe pericardial effusion were confirmed by chest computed tomography and/or pericardial ultrasound.

**Interventions:**

Low-dose colchicine (0.5 mg) was administered to each patient once a day for at least 3 months, in addition to prompt pericardiocentesis drainage, to address recurrent pericardial effusion. During colchicine treatment, liver and kidney function and routine blood test results were closely monitored for side effects.

**Outcomes:**

After 1 to 2 months of treatment, the recurrent pericardial effusion in all 3 patients was largely absorbed, their symptoms were greatly improved, and no side effects were observed. During colchicine treatment, liver and kidney function, myocardial enzyme levels, and routine blood tests showed no abnormalities.

**Lessons:**

More cases and longer follow-up periods are needed to confirm the efficacy and safety of low-dose colchicine for recurrent pericardial effusion in elderly patients. Interactions between colchicine and other medications also require further study, especially in elderly patients (≥80 years) with chronic diseases.

## 1. Introduction

The incidence of pericardial effusion in elderly patients is relatively high, and its clinical presentation is often atypical, leading to misdiagnoses or missed diagnoses.^[1]^ Due to physiological decline and the presence of various underlying diseases, the diagnosis and treatment of pericardial effusion in elderly patients face challenges, often accompanied by higher risks of complications and poor prognosis.^[2]^ Treatment of recurrent pericardial effusion is usually complex, with conventional methods such as puncture drainage and drug therapy showing limited effectiveness and high recurrence rates.^[3]^ This paper aimed to explore the application of low-dose colchicine in 3 elderly patients with recurrent pericardial effusion. As an anti-inflammatory drug, low-dose colchicine is a relatively new area of treatment for recurrent pericardial effusion in elderly patients, with limited related literature. Therefore, this study aimed to provide a new and safe treatment approach for recurrent pericardial effusion in elderly patients.

## 2. Case presentation

**Figure 1.**
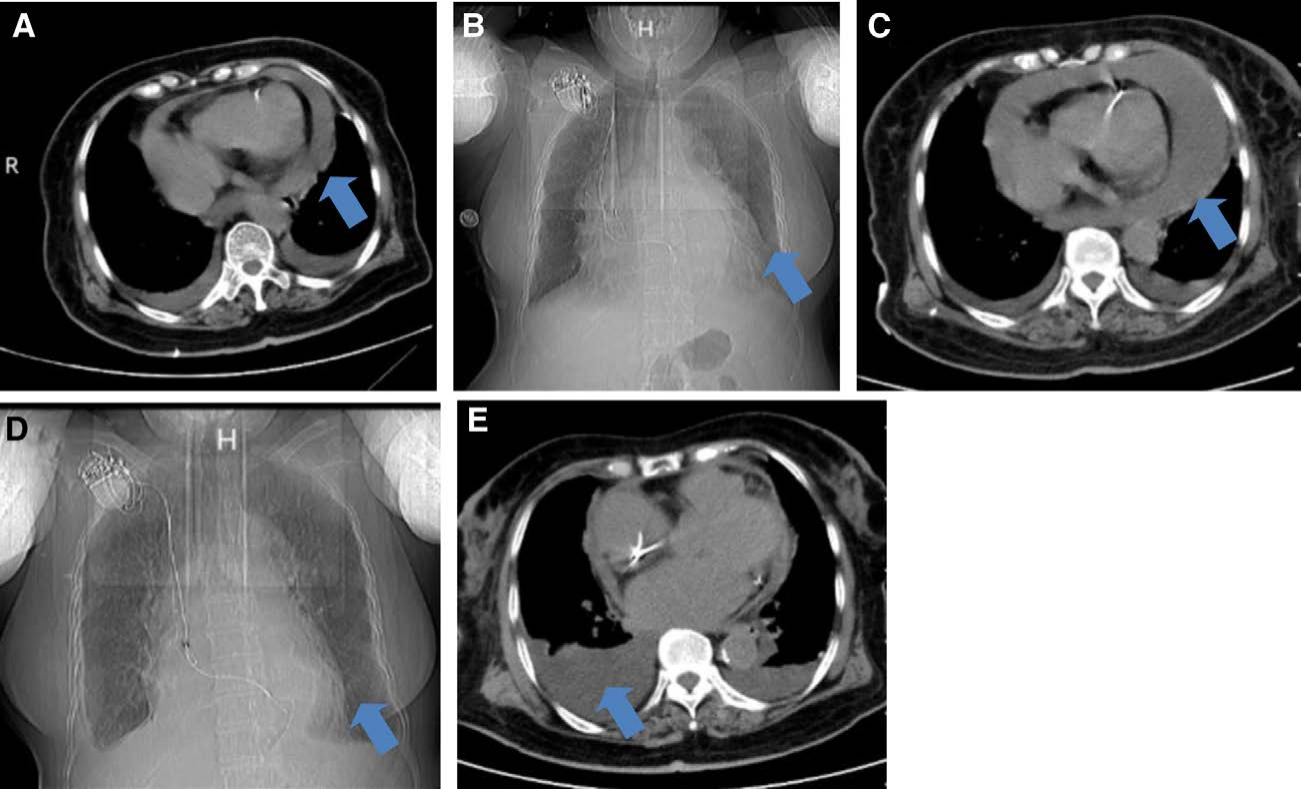
(A and B) First hospitalization chest CT showing PE and pleural effusion; (C) significant increase in pericardial effusion upon readmission. (D and E) Third hospitalization chest CT showing significant reduction in PE and right-sided pleural effusion. CT = computed tomography, PE = pericardial effusion.

*Case 1*: An 82-year-old female has undergone permanent pacemaker implantation over 20 years, accompanied by both diabetes and osteoporosis. She began experiencing paroxysmal atrial fibrillation (AF) about 3 years and started taking rivaroxaban 10 mg qd. Since September 2024, she has been hospitalized due to recurrent shortness of breath and edema. The patient’s brain natriuretic peptide was 220 pg/mL (0–100 pg/mL), new-onset pericardial and pleural effusion was found (Fig. 1A and B). After draining the pleural fluid, her symptoms improved; however, the pleural effusion quickly re-accumulated, and the pericardial effusion increased. With the patient’s consent, we performed bedside ultrasound-guided drainage of 500 to 600 mL of yellow pleural fluid daily for 3 days and 200 to 300 mL of bloody pericardial effusion for 3 days. Pericardial fluid analysis did not reveal any significant abnormalities. The pericardial effusion had basically disappeared, and the patient was discharged in the absence of pericardial effusion. However, the patient was readmitted with generalized edema and shortness of breath for 2 months. At this time, pericardial effusion (PE) increased significantly, with a large amount of left-sided pleural effusion (Fig. 1C). Similarly, we performed pericardial puncture drainage, which yielded 400 mL of bloody fluid over 4 days (100–200 mL daily). Tumor markers of pericardial fluid analysis were normal except for a slightly high CA125 level, and other tests including adenosine deaminase, lactate dehydrogenase, and inflammatory hmarkers were normal. Gastroscopy and enhanced abdominal computed tomography (CT) revealed no abnormalities. We collected samples for pathological testing, which revealed no abnormal exfoliated cells. Considering the unclear cause of the recurrent middle-large PE and the patient’s refusal for further examination at a higher-level hospital, we started low-dose colchicine (0.5 mg qd). One month later, during outpatient follow-up, the patient had unilateral pleural effusion, but the pericardial effusion had resolved (Fig. 1D and E), and she had no shortness of breath or edema, with normal liver and kidney function and electrolytes.

**Figure 2.**
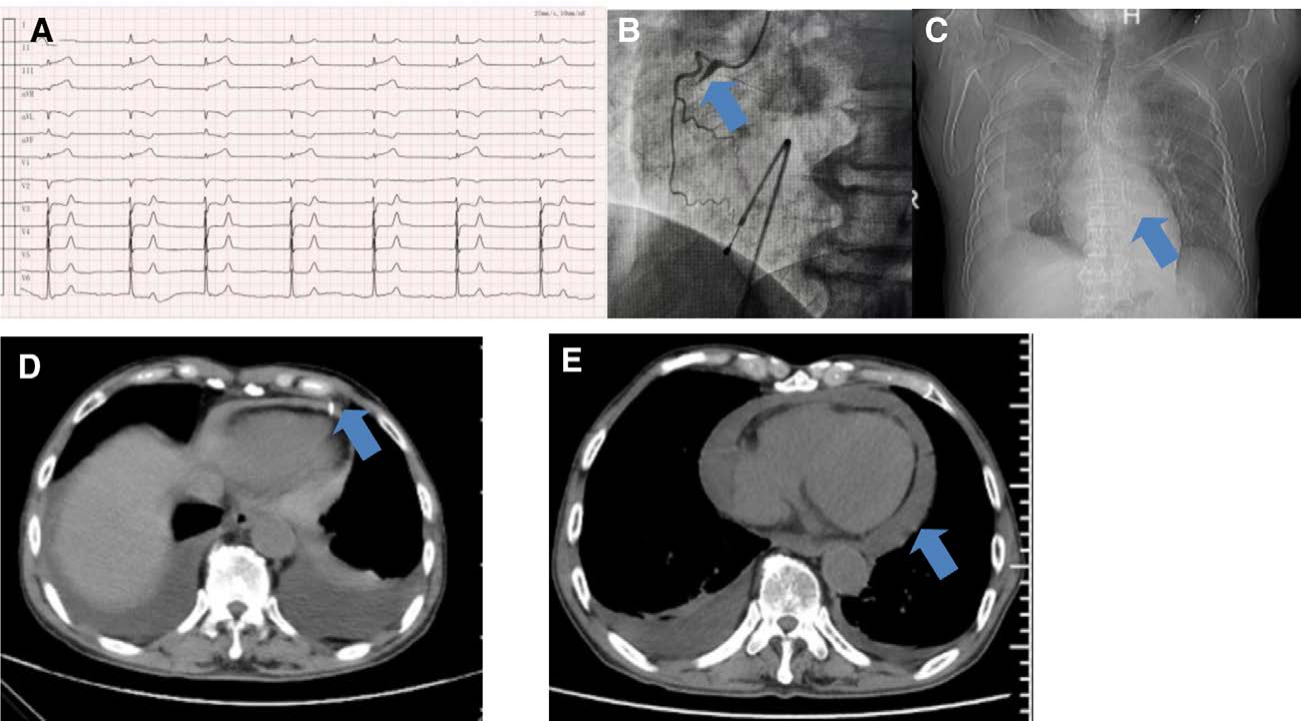
(A) Junctional rhythm (40 bpm) with ST-segment elevation in leads II, III, and aVF leads; (B) coronary angiography under temporary pacing showing complete occlusion of the proximal right coronary artery; (C and D) pericardial catheter placement. (E) Chest CT showing recurrence of pericardial effusion. CT = computed tomography.

*Case 2*: An 81-year-old male, with a history of hypertension and mild renal insufficiency was admitted because of chest tightness with dizziness and sweating for 28 hours. Upon admission, his blood pressure was 86/51 mm Hg, lethargic, and stable respiration, with a heart rate of 40 bpm. Electrocardiogram showed atrial rhythm with ST-segment elevation in leads II, III, and aVF with low voltage in the limb leads (Fig. 2A). Troponin was 18.5 ng/mL (0–0.1), myocardial enzymes 74.5 ng/mL (0–5), and blood gas analysis showed lactate at 2.9 mmol/L (1.0–1.7); ALT, 420 IU/L; AST, 590 IU/L; and creatinine, 227 µmol/L. A temporary pacemaker was implanted and coronary angiography revealed complete occlusion of the proximal right coronary artery (Fig. 2B), followed by revascularization. The patient had a heavy thrombus burden and was treated with anticoagulation and antiplatelet therapies postoperatively. On the second day, when the temporary pacemaker was removed, the patient experienced sudden loss of consciousness, with upward eye movement and profuse sweating. Bedside monitoring showed sinus rhythm, cardiac tamponade was considered, and bedside ultrasound revealed a large pericardial effusion with slight echogenic fluid, likely a blood clot. Norepinephrine (8 mg) was administered via a micro-pump, 250 mL of non-coagulated blood was drained from the pericardial cavity under bedside ultrasound guidance, and the patient’s vital signs gradually stabilized. During hospitalization, a chest CT was performed (Fig. 2C and D, showing pericardial catheter placement), and the patient was discharged after the pericardial effusion disappeared on ultrasonography. Two weeks later, a chest CT (Fig. 2E) showed more effusion than before, the patient showed moderate shortness of breath, a low-dose colchicine (0.5 mg qd) was administered for her PE. One and a half months later, pericardial ultrasonography revealed no PE.

**Figure 3.**
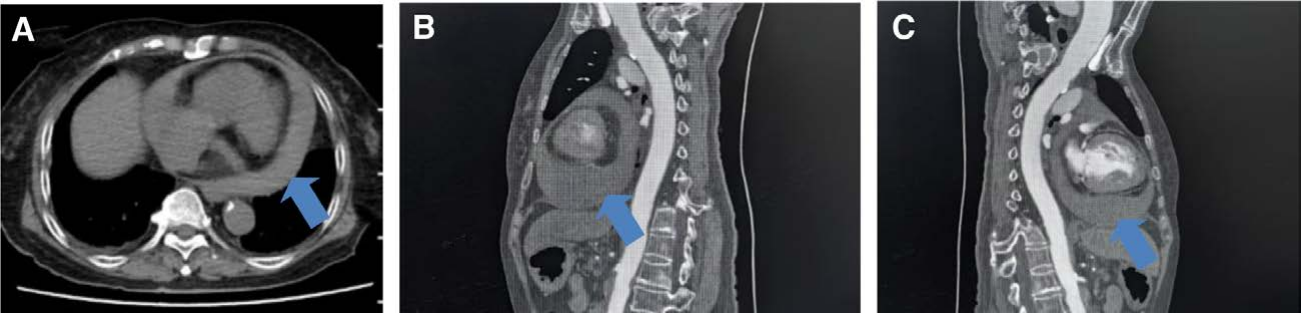
(A–C) Plain scan and sagittal view showing pericardial effusion.

*Case 3*: An 82-year-old female, with diabetes, paroxysmal AF, and ischemic heart disease was admitted to the emergency department because of dizziness for 2 hours and 1 episode of syncope. The patient complained no chest tightness, pain, fever, headache, abdominal pain, or vomiting. Physical examination revealed a blood pressure of 76/51 mm Hg, a respiratory rate of 16 bpm, and an oxygen saturation of 85%. She appeared to be conscious and normal responses, no abnormalities were found in the lung examination, low heart sounds, irregular heartbeat, lactate at 3.0 mmol/L, oxygen partial pressure at 72 mm Hg, and normal myocardial enzymes and troponin. D-dimer was 2.8 mg/L (0–0.5), and cranial CT showed no abnormalities. Enhanced chest CT indicated pericardial effusion (Fig. 3A–C), ruling out dissection or pulmonary embolism. Ultrasound-guided pericardial puncture drained 300 mL of hemorrhagic effusion, and the patient’s blood pressure quickly increased to 133/80 mm Hg. Laboratory tests of pleural fluid revealed no significant abnormalities. Half a month after discharge, recurrent pericardial effusion was found due to her breathing difficulty, low-dose colchicine (0.5 mg) was prescribed for 2 months, her PE nearly disappeared, and her liver and kidney function, myocardial enzymes, and routine blood tests were normal.

## 3. Discussion

Recurrent pericardial effusion refers to the repeated accumulation of fluid in the pericardial cavity, which is commonly observed in patients with various cardiovascular diseases, especially among elderly patients. Clinical manifestations of recurrent pericardial effusion include chest pain, shortness of breath, and palpitations, which often require repeated imaging assessments and treatments.^[4]^ Understanding the occurrence of pericardial effusion in the real world is challenging because of the significant variations in incidence across different countries and regions. The etiology is largely related to the local epidemiological characteristics of diseases; for instance, tuberculosis is the most common cause in sub-Saharan Africa. In developed countries, the most common causes of pericardial effusion include infections (15–30%), tumors (10–25%), iatrogenic causes (15–20%), and autoimmune/inflammatory diseases (5–10%); over 50% of cases in the Western world are nonspecific. Many patients with small-to-moderate pericardial effusion are asymptomatic and are thus not recorded.^[5]^ Elderly patients (≥80 years) are more likely to develop pericardial effusion due to further decline in physiological function, reduced immune function, and more pronounced issues such as infections, decline in heart and kidney function, and chronic inflammation, leading to a significantly increased recurrence rate of pericardial effusion.^[6]^

Elderly patients face more challenges when dealing with recurrent pericardial effusion. First, elderly patients often have multiple underlying diseases that impair cardiac function, further exacerbating pericardial effusion.^[7]^ Secondly, elderly patients may respond differently to medications than younger patients with a higher risk of side effects.^[8]^ Therefore, treatment plans for this group should be more individualized and cautiously evaluated to ensure safety and efficacy.

Among the various medications used to treat recurrent pericardial effusion, colchicine has garnered attention because of its unique anti-inflammatory properties. Colchicine is a traditional anti-inflammatory drug initially used to treat gout and familial Mediterranean fever, and recent studies have shown its increasing potential in cardiovascular diseases.^[9]^ Colchicine reduces the assembly of microtubules and decreases the release of inflammatory mediators, thereby alleviating pericardial inflammation and preventing recurrence of pericardial effusion to some extent.^[10]^ These characteristics suggest that colchicine is promising for the treatment of recurrent pericardial effusion in elderly patients. Colchicine has also been found to significantly reduce the release of various inflammatory factors, including tumor necrosis factor-α and interleukin-1β, which play important roles in inflammatory diseases such as pericardial effusion.^[11]^

Studies have indicated that colchicine can effectively reduce pericardial effusion and improve the functional status of the pericardium.^[12]^ It helps restore the normal structure and function of the pericardium by inhibiting the infiltration of inflammatory cells and fibrotic process in the pericardial tissue. Moreover, colchicine improves the microenvironment within the pericardium and lowers the levels of inflammatory factors, thus further promoting healing and functional recovery of the pericardium.^[13]^ This protective effect on pericardial tissue provides a theoretical basis and clinical support for the application of colchicine in elderly patients with recurrent pericardial effusion.

As an anti-inflammatory drug, colchicine has received increasing attention in the treatment of recurrent pericardial effusion in recent years. Multiple clinical trials have shown that colchicine significantly reduced the incidence of recurrent pericardial effusion. A retrospective study of patients with pericarditis also emphasized the effectiveness of colchicine in reducing the risk of pericardial effusion recurrence, with a significantly lower recurrence rate in the colchicine group than in patients not using the drug.^[14]^ This also provides clinical evidence for the application of colchicine in recurrent pericardial effusion.

The efficacy and safety of colchicine in the treatment of recurrent pericardial effusion have garnered much attention. Elderly patients face multiple comorbidities such as coronary heart disease, chronic kidney disease, diabetes, and diseases of the blood or digestive system. For instance, the patient in Case 1 had chronic heart failure, pacemaker implantation, type 2 diabetes, and severe osteoporosis; the patient in Case 2 had coronary heart disease and acute myocardial infarction, with the patient admitted in a state of shock and severe liver and kidney dysfunction; and Case 3 had low body weight, diabetes, and persistent AF. These patients often require statins or other medications, particularly in the elderly population, which may affect liver and kidney functions, especially considering the risk of muscle injury.^[15]^ Although colchicine has shown good safety in treating pericarditis and pericardial effusion, common adverse reactions include mild gastrointestinal discomfort with a low incidence of serious adverse events.^[16]^ However, considering the uniqueness of elderly patients, the characteristics of comorbid diseases, and the risks or side effects of using steroids or nonsteroidal anti-inflammatory drugs, individualized dosage adjustments are particularly important. We recommend that a daily dose of colchicine (0.5 mg) for elderly patients is reasonable, and clinicians should dynamically follow up on patient symptoms, monitor side effects, and regularly assess liver and kidney function and other physiological indicators to ensure drug safety and efficacy.

Additionally, all 3 patients in this report had hemorrhagic pericardial effusion drained through pericardiocentesis, and all cases involved anticoagulant medication. In Case 1, rivaroxaban 10 mg qd was administered for many years because of a previous minor cerebral infarction. Case 2 was understandable, as the right coronary artery had long-term occlusion with a heavy thrombus burden, and postoperatively, we used rivaroxaban 15 mg qd combined with clopidogrel 75 mg qd. The patient experienced pericardial bleeding due to cardiac perforation after the temporary pacemaker was removed; however, timely and effective pericardial drainage and subsequent management led to a very good prognosis. Case 3 involved a small elderly woman with persistent AF who was also taking rivaroxaban 10 mg qd for anticoagulation. The admission was due to syncope, and it could not be determined whether rivaroxaban caused hemorrhagic pericardial effusion; however, it is certain that this admission was due to cardiac tamponade. Upon discharge, all 3 elderly patients had acute cases, and during hospitalization, examinations and pericardial effusion tests did not reveal any evidence of viral infections, tuberculosis, rheumatic autoimmune diseases, or malignant tumors. We reviewed the literature and found that colchicine is effective in treating hemorrhagic pericardial effusion.^[17,18]^ However, its effectiveness in preventing the recurrence or progression of pericardial effusion remains unknown, as the hemorrhagic pericardial effusion in Cases 1 and 3 did not have a definitive relationship with anticoagulant treatment; thus, rivaroxaban was continuously administered in the outpatient setting alongside colchicine.

Low-dose colchicine not only reduces the recurrence of pericardial effusion but also minimizes side effects. In some populations, colchicine can reduce the occurrence of cardiovascular events. Therefore, colchicine is considered a safe and effective treatment for recurrent pericardial effusion.

Short-term observations indicate that low-dose colchicine treatment for recurrent pericardial effusion is safe and effective. However, ascertaining the safety and efficacy of long-term use requires further observation. Some causes of pericardial effusion are difficult to identify, as seen in our Cases 1 and 3, where no evidence of malignant pericardial effusion was found, but the etiology of the observed pericardial effusion was not very clear. Further observations are needed to determine whether low-dose colchicine is suitable for elderly patients with recurrent pericardial effusion of unknown origin. Interactions between colchicine and other medications also need to be taken seriously, especially in elderly patients (≥80 years) with chronic diseases requiring long-term treatment.

In conclusion, colchicine, as a traditional drug, has good application prospects in the prevention and treatment of cardiovascular diseases. Moreover, a meta-analysis by Xiong et al showed the effectiveness of colchicine.^[19]^ Following guidelines are needed to achieve more precise treatment plans for patient prognosis and quality of life. Individualized treatment is crucial for managing recurrent pericardial effusion. The 3 cases of elderly patients with recurrent pericardial effusion treated with low-dose colchicine not only effectively alleviated the symptoms of pericardial effusion, but also helped improve the patients’ quality of life, demonstrating good efficacy and safety in a short time. However, more extensive and robust clinical studies are needed to fully understand the long-term and side effects in the elderly population with recurrent pericardial effusion.

## Author contributions

**Data curation:** Peng-Ju Yan.

**Formal analysis:** Wen-Na Wang, Peng-Ju Yan.

**Investigation:** Wen-Na Wang, Hai-Ying Lu.

**Methodology:** Wen-Na Wang, Shan-Kuan Liu.

**Resources:** Hai-Ying Lu.

**Supervision:** Hai-Ying Lu, Shan-Kuan Liu.

**Validation:** Yang-Jian Zheng.

**Writing – original draft:** Yang-Jian Zheng.

**Writing – review & editing:** Yang-Jian Zheng.
